# Telomere length and appendicular lean mass over six years in older adults with overweight or obesity and metabolic syndrome: a prospective cohort study

**DOI:** 10.1007/s11357-026-02366-w

**Published:** 2026-06-20

**Authors:** Héctor Vázquez-Lorente, María Fernández de la Puente, Julio Plaza-Diaz, Nancy Babio, Montse Fitó, Miguel Ángel Martínez-González, Sonia García-Calzón, Amelia Marti del Moral, Jordi Salas-Salvadó

**Affiliations:** 1 Departament de Bioquímica I Biotecnologia, Alimentació, Nutrició, Desenvolupament i Salut Mental ANUT-DSM, Universitat Rovira I Virgili, Reus, Spain; 2 Institut de Recerca Biomèdica Catalunya Sud, Hospital Universitari Sant Joan de Reus, Reus, Spain; 3 Centro de Investigación Biomédica en Red Fisiopatología de La Obesidad y La Nutrición (CIBEROBN), Institute of Health Carlos III, Madrid, Spain; 4 Unit of Cardiovascular Risk and Nutrition, Hospital del Mar Research Institute (IMIM), Barcelona, Spain; 5 Department of Preventive Medicine and Public Health, Instituto de Investigación Sanitaria de Navarra (IdiSNA), University of Navarra, Pamplona, Spain; 6 Department of Nutrition, Harvard T.H. Chan School of Public Health, Boston, MA USA; 7 Department of Nutrition, Food Sciences and Physiology, Center for Nutrition and Research, Institute of Nutrition and Health, University of Navarra, Instituto de Investigación Sanitaria de Navarra (IdiSNA), Pamplona, Spain; 8 Epigenetic and Diabetes Unit, Department of Clinical Sciences, Lund University Diabetes Center, Scania University Hospital, Malmö, Sweden

**Keywords:** Telomeres, Appendicular lean mass, Ageing, Older adults

## Abstract

Telomere length (TL) has been proposed as a marker of appendicular lean mass (ALM) decline; however, longitudinal evidence remains limited. We aimed to investigate the longitudinal association between TL and 6-year trajectories of ALM. This longitudinal study included 185 older adults aged 55–75 years with overweight or obesity and metabolic syndrome at baseline. Baseline TL was assessed using a monochrome multiplex real-time quantitative PCR. ALM from the four limbs was obtained at baseline, and after 1, 3, and 6 years of follow-up using dual-energy X-ray absorptiometry. ALM was also normalized for height, total body weight, or body mass index (BMI). Baseline longer TL in continuous was associated with higher ALM (between-group difference, 0.76; 95% CI, 0.12 to 1.40) and greater lean mass in the lower limbs (between-group difference, 0.50; 95% CI, 0.02 to 0.97), ALM to height ratio (between-group difference, 0.21; 95% CI, 0.02 to 0.41), and higher ALM to BMI ratio (between-group difference, 0.02; 95% CI, 0.01 to 0.04) after 6 years. When analyzed categorically, participants with baseline TL above the median exhibited higher ALM (between-group difference, 0.62; 95% CI, 0.04 to 1.20) and greater lean mass in the lower limbs (between-group difference, 0.49; 95% CI, 0.06 to 0.92) and higher ALM to BMI ratio (between-group difference, 0.02; 95% CI, 0.01 to 0.04) compared with those below the median after 6 years. Longer baseline TL was modestly associated with more favorable long-term ALM trajectories in older adults with overweight or obesity and metabolic syndrome, although these observational findings preclude causal inference and should not be interpreted as evidence that telomere biology directly contributes to muscle aging.

**Supplementary information:**

The online version contains supplementary material available at https://doi.org/10.1007/s11357-026-02366-w.

## Introduction

Population aging is a global demographic phenomenon, with a sustained increase in both the absolute number and proportion of older adults worldwide [1]. This demographic shift is accompanied by a higher prevalence of age-related conditions, particularly those affecting the musculoskeletal system. Among these, the progressive decline in appendicular lean mass (ALM) is strongly associated with impaired physical function, increased risk of falls and fractures, metabolic disturbances, and loss of independence [2]. Although closely linked to advancing age, ALM decline is also influenced by chronic systemic diseases as well as environmental and genetic factors that may accelerate its progression [3]. Elucidating the biological mechanisms underlying ALM deterioration has therefore become central to research on the hallmarks of aging and their contribution to functional decline in later life [4].

Telomeres are repetitive, non-coding DNA sequences that safeguard chromosomal stability. Their progressive shortening, accelerated by cell division and oxidative stress, is a recognized hallmark of aging and contributes to cellular senescence and tissue dysfunction [5, 6]. Telomere length (TL) varies markedly among individuals of the same chronological age, reflecting genetic, epigenetic, and environmental influences, and is therefore considered an indicator of biological age [7]. Although relatively stable in early life, TL declines with advancing age and has been associated with years of healthy living, supporting its role as a potential biomarker of healthy aging [8]. Despite growing interest in identifying reliable markers of biological age, few measures have demonstrated consistent clinical applicability [9]. Whether as a driver or mediator, telomere dysfunction is increasingly recognized as an integral component of the molecular processes underlying aging and age-related disease [10].

As telomere attrition and ALM decline are both fundamental components of the aging process, a potential biological association between TL and ALM has been suggested [11]. The coexistence of shorter TL and lower ALM may reflect an increased vulnerability to unhealthy aging and age-related diseases [12] Conversely, preservation of ALM may contribute to attenuate telomere attrition, and vice versa [13]. However, evidence in older adults remains inconsistent. Cross-sectional evidence in older adults is mixed. Some studies have reported positive associations between TL and ALM [11, 14] or appendicular lean mass index (ALMI) [11, 15], whereas others have not observed significant associations with ALMI [16, 17]. Longitudinal evidence is sparse and the unique available prospective study in older Chinese adults did not identify association between baseline TL and long-term changes in ALMI [16]. These discrepancies may be explained by differences in age, study design, population characteristics, and definitions used for preserved versus low muscle mass [16, 17].

Given the clinical importance of identifying accessible biomarkers of ALM decline and the recognized role of telomere attrition in biological aging, further longitudinal research is therefore warranted to clarify this association [11]. Based on this rationale, the present study aimed to investigate the association between baseline TL and 6-year trajectories of ALM and ALMI in older Caucasian Mediterranean adults with overweight or obesity and metabolic syndrome.

## Materials and methods

### Study design

**Fig. 1:**
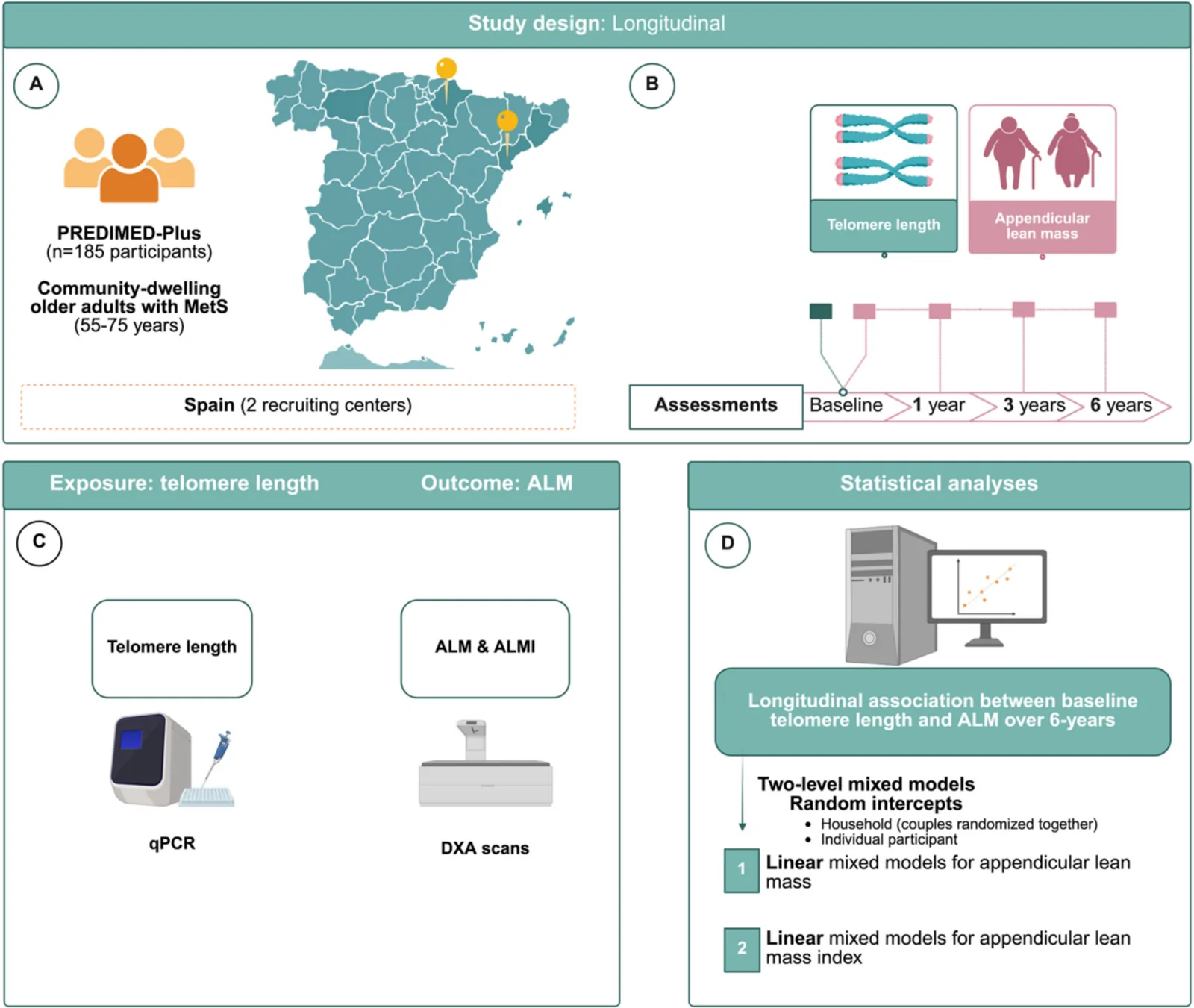
Study design. Abbreviations: ALM, appendicular lean mass; ALMI, appendicular lean mass index; DXA, dual-energy x-ray absorptiometry; MetS, metabolic syndrome; PREDIMED-Plus, Prevención con Dieta Mediterránea–Plus

This study was a prospective observational analysis nested within the framework of the PREDIMED-Plus trial (Fig. 1) [18]. Briefly, PREDIMED-Plus is a multicenter, parallel-group, randomized, single-blind clinical trial designed to evaluate the long-term effects of a comprehensive lifestyle intervention including an energy-reduced Mediterranean diet, physical activity promotion, and behavioral support for weight-loss (intervention group), compared with general recommendations to follow an ad libitum Mediterranean diet (control group). The main objectives of the trial are the primary prevention of cardiovascular disease and long-term body weight-loss maintenance. Details of the PREDIMED-Plus protocol have been published elsewhere [19, 20], and are available online at https://www.predimedplus.com/. Ethical approval was obtained from the institutional review boards of all participating centers, and all participants provided written informed consent. The trial was registered in 2014 at ISRCTN.com (ISRCTN89898870).

### Participants

**Fig. 2:**
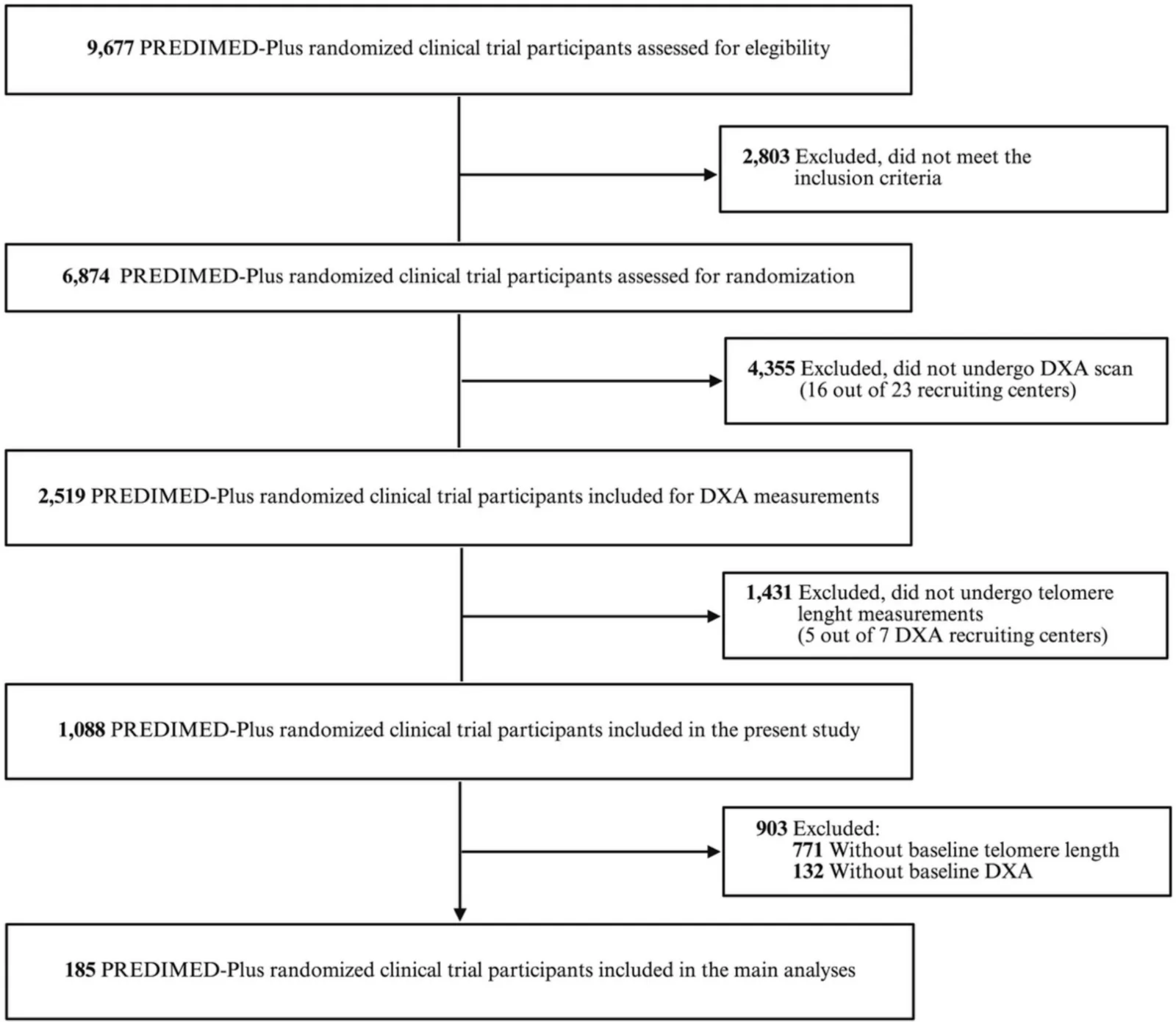
Flow chart of the study population. Abbreviations: DXA, dual-energy x-ray absorptiometry; PREDIMED-Plus, Prevención con Dieta Mediterránea–Plus

Participants were community-dwelling adults aged 55–75 years with overweight or obesity (body mass index (BMI) 27 to 40 kg/m^2^) and metabolic syndrome (Fig. 1A) [21]. Metabolic syndrome was identified according to harmonized criteria established by the International Diabetes Federation, and the American Heart, Lung, and Blood Institute, requiring the presence of at least three of the following components: elevated blood pressure (≥ 130/85 mmHg), elevated plasma triglycerides (≥ 150 mg/dL), low plasma high-density lipoprotein cholesterol (HDL [< 40 mg/dL for males or < 50 mg/dL for females]), elevated fasting blood glucose (≥ 100 mg/dL), and central obesity (waist circumference ≥ 102 cm for males or ≥ 88 cm for females) [21]. Exclusion criteria included the following: (I) unwillingness to give written informed consent; (II) institutionalization; (III) pre-existing cardiovascular disease, psychiatric disorders, or bowel diseases; (IV) use of weight loss medication; and (V) inability to adhere to the intervention due to religious, dietary, or medical restrictions among others. Between October 2013 and December 2016, a total of 6874 eligible participants were randomly assigned in a 1:1 ratio to either the intervention group or the control group. Randomization was blinded to all research staff and principal investigators. In cases where two participants from the same household enrolled, randomization was performed by cluster, considering the household as the unit of allocation. For the purposes of the present study, we used data from a subsample of participants who underwent dual-energy X-ray absorptiometry (DXA) measurements in 7 of the 23 recruiting centers having access to DXA scanners (DXA sub-study). Among 2,519 eligible participants in this sub-study, 2 of the 7 recruiting centers having access to DXA scanners had available baseline measurements of TL in addition to DXA assessments (Fig. 2).

### Telomere length assessment

Blood samples were obtained after an overnight fast. Buffy coat fractions were isolated and stored at −80 °C until DNA extraction. Genomic DNA was extracted from frozen buffy coat samples using the Maxwell RSC Blood DNA Kit (Promega, Madison, WI, USA) on the Maxwell RSC Instrument, following the manufacturer’s instructions. Relative leukocyte TL was determined at baseline using a monochrome multiplex real-time quantitative PCR (MMqPCR) assay based on the method described by Cawthon [22]. In each reaction, telomere repeat copy number (T) and a single-copy reference gene (albumin; S) were amplified simultaneously. All samples were analyzed in triplicate in 384-well plates using a CFX384 Touch Real-Time PCR Detection System (Bio-Rad, Hercules, CA, USA).

PCR reactions were prepared using the QuantiTect SYBR Green PCR Kit (Qiagen, Hilden, Germany) in a final volume of 10 µL containing 10 ng of genomic DNA, telomere and albumin primers, and nuclease-free water. Primer sequences were as follows: telg, 5′-ACACTAAGGTTTGGGTTTGGGTTTGGGTTTGGGTTAGTGT-3′; telc, 5′-TGTTAGGTATCCCTATCCCTATCCCTATCCCTATCCCTAACA-3′; albu, 5′-CGGCGGCGGGCGGCGCGGGCTGGGCGGAAATGCTGCACAGAATCCTTG-3′; and albd, 5′-GCCCGGCCCGCCGCGCCCGTCCCGCCGGAAAAGCATGGTCGCCTGTT-3′. Each plate included a seven-point standard curve generated from reference DNA using a twofold serial dilution (150 to 2.34 ng/mL), negative controls, and calibrator samples to monitor inter-plate variability. Assay performance was assessed according to predefined criteria, including standard-curve linearity, amplification efficiency, and replicate agreement. Intra-assay and inter-assay coefficients of variation were calculated, and samples not meeting quality thresholds were repeated. Only runs with standard curve linearity *R*^*2*^ > 0.99 were accepted. TL was calculated as the telomere-to-single-copy gene (T/S) ratio using the standard curve method for relative quantification.

TL was measured in a subsample of 185 participants from the Reus and Pamplona centers of the PREDIMED-Plus study at baseline, based on prior experience in TL assessment in human samples and laboratory resource availability. These two centers were the only sites participating in this sub study (Fig. 2).

### Appendicular lean mass assessment

At baseline, and after 1, 3, and 6 years of follow-up, height (cm) was measured in light clothing and without shoes using a calibrated scale and wall-mounted stadiometer. Body composition was assessed by dual-energy X-ray absorptiometry using Lunar iDXA or DXA Lunar Prodigy Primo devices (GE Healthcare, Madison, WI). Scans were performed by trained radiology technicians following standardized manufacturer protocols for participant positioning. Each DXA device was calibrated daily according to manufacturer guidelines. Total body weight and regionally distributed lean mass in arms and legs were obtained. ALM (kg) was calculated as the sum of lean mass from the four limbs. BMI was calculated as (total body weight [kg]/height [m]^2^). The following ALMI was subsequently computed: the ALM to height ratio (ALM [kg]/height^2^ [m^2^]), the ALM to total body weight ratio (ALM [kg]/total body weight [kg] × 100), and the ALM to BMI ratio (ALM [kg]/BMI [kg/m^2^] × 100) (Fig. 1C).

### Covariate assessments

Baseline covariates included sociodemographic and lifestyle information. Data on age, sex, education level, marital, and smoking status were collected through administered questionnaires. Physical activity was estimated using the validated REGICOR (Registre Gironí del Cor) Short Physical Activity Questionnaire for adults adapted from the Minnesota Leisure Time Physical Activity Questionnaire (MLTPAQ) [23], whereas sedentary behavior was assessed using the validated Spanish version of the Nurses’ Health Study questionnaire [24]. Medical history, including type 2 diabetes, hypercholesterolemia, and individual components of the metabolic syndrome (hypertension, hypertriglyceridemia, low-HDL cholesterol, hyperglycemia, and central obesity), as well as medication use, was obtained from self-reports or extracted from medical records. Habitual dietary intake was assessed at baseline using a validated 143-item semi-quantitative food frequency questionnaire (FFQ) [25]. Total energy and alcohol intake were estimated using Spanish food composition tables [26].

### Statistical analyses

Main analyses were performed in the evaluable population, including all participants with complete baseline data on exposure, outcomes, and covariates, as well as those who were lost to follow-up at 1, 3, or 6 years.

Baseline characteristics were summarized for the total sample and according to categories of baseline TL. Continuous variables were expressed as means ± standard deviations (SDs), and categorical variables as counts (percentages). Differences across baseline TL categories were examined using one-way analysis of variance (ANOVA) for continuous variables and the chi-square test for categorical variables.

Two-level linear mixed-effects models were fitted to assess the association between baseline TL, modeled as a continuous variable and by predefined categories (< median and ≥ median), and trajectories of ALM or ALMI over 1, 3, and 6 years of follow-up with random intercepts at the household cluster level (as couples from the same household were randomized together), and at the individual participant levels (Fig. 1D). In both cases, an interaction term between baseline TL and time, age (years), sex (male or female), recruiting center (Reus or Pamplona), intervention group (control or intervention), education level (primary or less, secondary, or college), marital status (single, divorced or separated, married, or widower), smoking status (former or never), type 2 diabetes prevalence (yes or no), hypertension prevalence (yes or no), hypercholesterolemia prevalence (yes or no), physical activity (metabolic equivalents in minutes per day), sedentary time (hours per day), alcohol consumption in grams per day (and adding the quadratic term), and total energy intake (kilocalories per day) were included as fixed effects in the multivariable-adjusted models. Results from the linear mixed models were reported as intergroup mean differences with their corresponding 95% confidence intervals (CIs).

A priori interaction analyses were conducted to test for potential effect modification in the association between baseline TL (as continuous) and ALM across baseline categories of age (< 65 vs. ≥ 65 years), sex (male or female), intervention group (control/intervention), type 2 diabetes prevalence (yes or no), smoking status (former or never), physical activity (< median vs. ≥ median), sedentary time (< median vs. ≥ median), alcohol consumption (< median vs. ≥ median), and energy intake (< median vs. ≥ median). Likelihood ratio test was used to evaluate the statistical significance of the interaction terms.

Sensitivity analyses were conducted by performing a complete-case analysis that excluded participants with missing ALM data at 1, 3, and 6 years of follow-up.

All statistical analyses were conducted using Stata/SE version 14.2 (StataCorp LLC, College Station, TX, USA) based on the PREDIMED-Plus study dataset updated to December 19, 2023. All graphs were plotted using GraphPad Prism software v.9.0 (GraphPad Software, San Diego, CA, USA). Statistical significance was defined as a two-tailed *P*-value < 0.05.

## Results

The number of participants with and without ALM data from centers equipped with DXA is presented in Table S1. The number of participants with and without TL measurements from centers with access to TL measurements is shown in Table S2. Baseline characteristics of participants selected for DXA assessment, compared with those not selected from the overall cohort, are detailed in Table S3. Table 1 summarizes the baseline characteristics of the study population stratified by median baseline TL. A total of 185 participants, with a mean (SD) age of 65.2 (5.1) years (80 [43.2%] females), were included in the current analysis. All participants were Caucasian and fulfilled the diagnostic criteria for metabolic syndrome.

**Table 1:** Baseline characteristics of the PREDIMED-Plus participants

Characteristic	Total subsample of PREDIMED-Plus (*n* = 185)	Categories of telomere length		*P*-value
		< Median (*n* = 93)	≥ Median (*n* = 92)	
Sociodemographic variables				
Age, mean (SD), years	65.2 ± 5.1	65.6 ± 5.6	64.8 ± 4.6	0.232
Female, No. (%)	80 (43.2)	41 (44.1)	39 (42.4)	0.816
Education level, No. (%)				
Primary or less	90 (48.7)	43 (46.3)	47 (51.1)	0.747
Secondary	61 (33.0)	33 (35.5)	28 (30.4)	
College	34 (18.4)	17 (18.3)	17 (18.5)	
Civil status, No. (%)				
Single, divorced or separated	17 (9.2)	10 (10.8)	7 (7.6)	0.723
Married	153 (82.7)	75 (80.7)	78 (84.8)	
Widower	15 (8.1)	8 (8.6)	7 (7.6)	
Ethnicity				
Caucasian	185 (100)	93 (100)	92 (100)	–
African	0 (0)	0 (0)	0 (0)	
Asian	0 (0)	0 (0)	0 (0)	
Hispanic or Latino	0 (0)	0 (0)	0 (0)	
Other	0 (0)	0 (0)	0 (0)	
Disease presence or medication usage at recruitment				
Type 2 diabetes, No. (%)	55 (29.7)	28 (30.1)	27 (29.4)	0.910
Hypercholesterolemia, No. (%)	125 (67.6)	65 (69.9)	60 (65.2)	0.497
Metabolic syndrome, No. (%)	185 (100)	93 (100)	92 (100)	–
High blood pressure, No. (%)	151 (81.6)	81 (87.1)	70 (76.1)	0.053
High triglycerides, No. (%)	83 (44.9)	42 (45.2)	41 (41.6)	0.935
Low HDL-cholesterol, No. (%)	63 (34.1)	35 (37.6)	28 (30.4)	0.302
Hyperglycemia, No. (%)	105 (56.8)	51 (54.8)	54 (58.7)	0.596
Central obesity, No. (%)	147 (79.5)	77 (82.8)	70 (76.1)	0.259
Medication use, No. (%)				
Insulin or other antidiabetic drugs	44 (23.8)	23 (24.7)	21 (22.8)	0.761
Antihypertensive agents	150 (81.1)	76 (81.7)	74 (80.4)	0.823
Statins or other hypolipidemic drugs	98 (53.0)	54 (58.1)	44 (47.8)	0.163
Lifestyle variables				
Physical activity, mean (SD), METs/min/d	422.0 ± 389.0	419.6 ± 389.4	424.4 ± 390.8	0.934
Sedentary time, mean (SD), h/d	5.6 ± 1.8	5.5 ± 1.7	5.7 ± 1.9	0.448
Smoking status, No. (%)				
Former smoker	93 (50.3)	50 (53.8)	43 (46.7)	0.339
Never smoker	92 (49.7)	43 (46.2)	49 (53.3)	
Dietary variables				
Alcohol intake, mean (SD), g/d	11.7 ± 13.4	10.5 ± 12.6	12.9 ± 14.1	0.234
Energy intake, mean (SD), kcal/d	2437 ± 523	2413 ± 501	2461 ± 546	0.538
Appendicular lean mass				
Appendicular lean mass, mean (SD), kg	22.3 ± 0.4	22.5 ± 5.2	22.1 ± 0.5	0.652
Upper limbs lean mass, mean (SD), kg	6.1 ± 1.7	6.2 ± 1.8	6.0 ± 1.6	0.529
Lower limbs lean mass, mean (SD), kg	16.2 ± 3.4	16.3 ± 3.7	16.1 ± 3.2	0.742
ALM to height ratio, mean (SD)	6.8 ± 1.2	6.8 ± 1.3	6.7 ± 0.1	0.847
ALM to total body weight ratio, mean (SD)	26.0 ± 3.9	26.3 ± 4.0	25.8 ± 3.8	0.409
ALM to BMI ratio, mean (SD)	0.7 ± 0.2	0.7 ± 0.2	0.7 ± 0.2	0.288

*ALM* appendicular lean mass, *BMI* body mass index, *HDL* high-density lipoprotein cholesterol, *METs* metabolic equivalents, *PREDIMED-Plus* Prevención con Dieta Mediterránea–Plus

Data are presented as *n* (%) or mean ± SD for categorical and continuous variables, respectively

Table 2 presents the associations between baseline TL and ALM over 6 years of follow-up. After multivariable adjustment, baseline longer TL, analyzed as a continuous variable, was associated with higher ALM (between-group difference, 0.76; 95% CI, 0.12 to 1.40) and greater lean mass in the lower limbs (between-group difference, 0.50; 95% CI, 0.02 to 0.97) after 6 years. When analyzed categorically, participants with baseline TL above the median exhibited higher ALM (between-group difference, 0.62; 95% CI, 0.04 to 1.20) and greater lean mass in the lower limbs (between-group difference, 0.49; 95% CI, 0.06 to 0.92) compared with those below the median after 6 years. These associations remained statistically significant in complete-case analyses. In complete-case sensitive analyses, statistically significant associations were also observed for upper limb lean mass both for continuous baseline TL (between-group difference, 0.51; 95% CI, 0.14 to 0.87) and for comparisons above versus below the median baseline TL (between-group difference, 0.56; 95% CI, 0.21 to 0.92) after 6 years (Table S4).

**Table 2:** Baseline telomere length and appendicular lean mass over 6 years^a^

Variable	Multivariable-adjusted model			
	Continuous	< Median	≥ Median	*P*-trend^b^
Appendicular lean mass (kg)				
Baseline	0 (Ref.)	0 (Ref.)	0 (Ref.)	0.081
Year 1 vs baseline	0.39 [–0.17 to 0.95]	0 (Ref.)	**0.57 [0.04 to 1.10]**	
Year 3 vs baseline	0.47 [–0.11 to 1.05]	0 (Ref.)	0.48 [–0.05 to 1.01]	
Year 6 vs baseline	**0.76 [0.12 to 1.40]**	0 (Ref.)	**0.62 [0.04 to 1.20]**	
Upper limbs lean mass (kg)				
Baseline	0 (Ref.)	0 (Ref.)	0 (Ref.)	0.261
Year 1 vs baseline	0.12 [–0.18 to 0.43]	0 (Ref.)	0.13 [–0.16 to 0.42]	
Year 3 vs baseline	0.29 [–0.03 to 0.61]	0 (Ref.)	**0.30 [0.01 to 0.59]**	
Year 6 vs baseline	0.25 [–0.09 to 0.60]	0 (Ref.)	0.11 [–0.20 to 0.43]	
Lower limbs lean mass (kg)				
Baseline	0 (Ref.)	0 (Ref.)	0 (Ref.)	0.068
Year 1 vs baseline	0.26 [–0.16 to 0.68]	0 (Ref.)	**0.43 [0.04 to 0.83]**	
Year 3 vs baseline	0.18 [–0.25 to 0.61]	0 (Ref.)	0.18 [–0.21 to 0.58]	
Year 6 vs baseline	**0.50 [0.02 to 0.97]**	0 (Ref.)	**0.49 [0.06 to 0.92]**	

*ALM* appendicular lean mass, *CI* confidence interval

^a^Two-level linear mixed models were fitted with random intercepts at the cluster family (as couples from the same household were randomized together) and individual participants to assess the relationship baseline telome length, modeled as a continuous variable and by predefined categories (< median and ≥ median) and appendicular lean mass (outcome) measured repeatedly over time (at each follow-up visit and for the overall follow-up period). Multivariable-adjusted models included fixed effects for an interaction term between baseline telomere length and time, as well as age (years), sex (male or female), recruiting center, intervention group (control or intervention), education level (primary or less, secondary, or college), marital status (single, divorced or separated, married, or widowed), smoking status (former, or never), diabetes prevalence (yes or no), hypertension prevalence (yes or no), hypercholesterolemia prevalence (yes or no), physical activity (metabolic equivalents in minutes per day), sedentary time (hours per day), alcohol consumption in grams per day (and adding the quadratic term), and total energy intake (kilocalories per day). Data are presented as mean differences [95% CI]. Significant values (*p* < 0.05) were highlighted in bold type

^b^*P*-value for the overall follow-up period

Table 3 shows the associations between baseline TL and ALMI over 6 years. In multivariable-adjusted models, continuous baseline longer TL was associated with higher ALM to height ratio (between-group difference, 0.21; 95% CI, 0.02 to 0.41) and higher ALM to BMI ratio (between-group difference, 0.02; 95% CI, 0.01 to 0.04) after 6 years. Participants with baseline TL above the median also demonstrated higher ALM to BMI ratio (between-group difference, 0.02; 95% CI, 0.01 to 0.04) compared with those below the median after 6 years. These associations were robust in complete-case analyses. Furthermore, in complete-case sensitivity analyses, continuous baseline TL was also associated with a higher ALM to total body weight ratio (between-group difference, 1.23; 95% CI, 0.35 to 2.10) after 6 years, whereas in categorical analyses, participants above the median baseline TL also had higher ALM to BMI ratio (between-group difference, 0.35; 95% CI, 0.13 to 0.58) and higher ALM to total body weight ratio (between-group difference, 1.39; 95% CI, 0.54 to 2.24) after 6 years (Table S4).

**Table 3:** Baseline telomere length and appendicular lean mass indexes over 6 years^a^

Variable	Multivariable-adjusted model			
	Continuous	< Median	≥ Median	*P*-trend^b^
Appendicular lean mass to height ratio				
Baseline	0 (Ref.)	0 (Ref.)	0 (Ref.)	0.114
Year 1 vs baseline	0.11 [− 0.06 to 0.28]	0 (Ref.)	**0.16 [0.01 to 0.32]**	
Year 3 vs baseline	0.14 [− 0.04 to 0.31]	0 (Ref.)	0.14 [− 0.02 to 0.31]	
Year 6 vs baseline	**0.21 [0.02 to 0.41]**	0 (Ref.)	0.17 [− 0.01 to 0.34]	
Appendicular lean mass to total body weight ratio				
Baseline	0 (Ref.)	0 (Ref.)	0 (Ref.)	0.144
Year 1 vs baseline	0.54 [− 0.11 to 1.19]	0 (Ref.)	**0.64 [0.03 to 1.25]**	
Year 3 vs baseline	0.23 [− 0.48 to 0.94]	0 (Ref.)	0.44 [− 0.22 to 1.11]	
Year 6 vs baseline	0.67 [− 0.06 to 1.40]	0 (Ref.)	0.61 [− 0.06 to 1.28]	
Appendicular lean mass to body mass index ratio				
Baseline	0 (Ref.)	0 (Ref.)	0 (Ref.)	0.084
Year 1 vs baseline	0.02 [− 0.01 to 0.03]	0 (Ref.)	**0.02 [0.01 to 0.04]**	
Year 3 vs baseline	0.01 [− 0.01 to 0.03]	0 (Ref.)	0.01 [− 0.01 to 0.03]	
Year 6 vs baseline	**0.02 [0.01 to 0.04]**	0 (Ref.)	**0.02 [0.01 to 0.04]**	

*ALM* appendicular lean mass, *CI* confidence interval

^a^Two-level linear mixed models were fitted with random intercepts at the cluster family (as couples from the same household were randomized together) and individual participants to assess the relationship baseline telome length, modeled as a continuous variable and by predefined categories (< median and ≥ median) and appendicular lean mass indexes (outcome) measured repeatedly over time (at each follow-up visit and for the overall follow-up period). Multivariable-adjusted models included fixed effects for an interaction term between baseline telomere length and time, as well as age (years), sex (male or female), recruiting center, intervention group (control or intervention), education level (primary or less, secondary, or college), marital status (single, divorced or separated, married, or widowed), smoking status (former or never), diabetes prevalence (yes or no), hypertension prevalence (yes or no), hypercholesterolemia prevalence (yes or no), physical activity (metabolic equivalents in minutes per day), sedentary time (hours per day), alcohol consumption in grams per day (and adding the quadratic term), and total energy intake (kilocalories per day). Data are presented as mean differences [95% CI]. Significant values (*p* < 0.05) were highlighted in bold type

^b^*P*-value for the overall follow-up period

A significant interaction by age was detected for the association between baseline TL (continuous) and ALM over 6 years (*P* = 0.001) (Fig. S1). No significant interactions were detected for sex, study arm, type 2 diabetes prevalence, smoking status, physical activity sedentary time, or energy intake. In age-stratified analyses, the positive association between baseline TL and ALM over 6 years persisted only among participants aged ≥ 65 years (Fig. S1).

## Discussion

This longitudinal analysis provides prospective evidence on the association between baseline leukocyte TL and ALM over 6 years in older adults with overweight or obesity and metabolic syndrome. Longer baseline TL was modestly but consistently associated with more favorable ALM trajectories after adjustment for a comprehensive set of potential confounders. Individuals with longer TL showed higher ALM values, largely driven by the lower-limb compartment. ALM values standardized to height or to BMI yielded the most consistent and clinically meaningful associations. Although the association appeared more evident among participants aged ≥ 65 years, these age-stratified findings should be interpreted cautiously because of the modest sample size and exploratory nature of subgroup analyses.

To date, no randomized intervention trials have directly examined whether modifying TL influences the preservation of ALM. Evidence is limited to observational studies, mostly cross-sectional studies suggesting a protective relationship between longer telomeres and greater ALM. For instance, in adults aged > 60 years in the UK, longer TL was associated with higher ALM [14]. Similarly, data from a German cohort of individuals aged 60–80 years reported that shorter TL was associated with lower ALM, particularly in the lower extremities [11]. The present study extends this literature by providing prospective data over 6 years, demonstrating a sustained positive association between longer TL and ALM in a European cohort, although the magnitude of the observed associations was modest.

Evidence regarding TL and ALMI remains heterogeneous. In cross-sectional analyses, a significant positive association between TL and ALM to BMI ratio was reported in the previously described German cohort of adults aged 60–80 years [11]. Similarly, among Italian adults aged ≥ 65 years, longer TL was positively associated with ALM to height ratio, suggesting a potential link between telomere biology and standardized measures of muscle mass [15]. Conversely, studies in older adults from China and UK did not observe significant associations between TL and ALM to height ratio [16, 17]. Notably, the Chinese cohort also included a 5-year longitudinal component and did not identify a significant prospective association [16]. In this context, our findings add longitudinal evidence supporting an association between longer TL and more favorable ALMI trajectories. The use of three standardization approaches improves interpretability: ALM to height ratio is widely used in sarcopenia definitions, whereas ALM to BMI and ALM to total body weight may be particularly informative in older adults with excess adiposity, in whom ALM to height ratio may underestimate low relative ALM [27–29].

From a geroscience perspective, the association between leukocyte TL and ALM may reflect shared biological aging pathways rather than a direct effect of leukocyte TL on skeletal muscle [30]. Age-related muscle decline is increasingly understood as a multifactorial process involving cellular senescence, satellite-cell dysfunction or exhaustion, impaired mitochondrial quality control, inflammaging, anabolic resistance, stem-cell dysfunction, and reduced regenerative resilience [6, 9, 31–33]. Telomere attrition may contribute to some of these processes by limiting proliferative capacity and promoting cellular senescence, while oxidative stress, chronic low-grade inflammation, metabolic impairment, and mitochondrial dysfunction can simultaneously accelerate telomere shortening and muscle deterioration. Nevertheless, our study cannot determine whether shorter leukocyte TL contributes to ALM decline, whether declining muscle health accelerates systemic biological aging, or whether both reflect shared upstream determinants [14, 33, 34]. Longitudinal studies incorporating repeated leukocyte TL measures, skeletal-muscle-specific biomarkers, inflammatory and mitochondrial markers, and functional outcomes are needed to clarify the directionality and potential mechanistic basis of these pathways [31].

Taken together, these findings suggest that leukocyte TL may provide limited complementary information on ALM trajectories, although its value as a predictive biomarker remains uncertain. In this population of older adults with overweight or obesity and metabolic syndrome, leukocyte TL may be better interpreted as a general marker of cumulative biological stress or multimorbidity burden than as a direct biomarker of skeletal muscle aging [35]. Established contributors to telomere attrition include smoking, obesity, psychological stress, physical inactivity, suboptimal dietary patterns, and exposure to air pollution [36–38]. Lifestyle interventions based on dietary improvement and physical activity may influence both TL dynamics and body composition. Therefore, TL assessment should not currently be proposed as a stand-alone clinical tool for identifying older adults at risk of muscle loss [11, 33, 34, 39, 40].

The present study has several methodological strengths. The analysis specifically targeted ALM rather than total lean mass, thereby reducing potential distortion from variability in noncontractile lean tissues. DXA-derived ALM has been validated against reference imaging techniques, including computed tomography and magnetic resonance imaging techniques [41], supporting its measurement validity. The longitudinal design allowed assessment of temporal associations over 6 years, with comprehensive adjustment for relevant confounders. In addition, analytical variability in TL quantification was minimized through the use of the MMqPCR method, which simultaneously amplifies telomeric repeats and a single-copy reference gene within the same reaction well, thereby enhancing precision. Limitations should also be acknowledged. Residual confounding and reverse causation cannot be fully excluded. Although multiple covariates were incorporated into the models, information on certain genetic and biological factors that could act as confounders was unavailable, limiting mechanistic inference. The external validity of the findings is restricted to older adults with overweight or obesity and metabolic syndrome and may be further limited by the availability of TL measurements in only two recruiting centers. Therefore, the association between leukocyte TL and ALM trajectories may differ in metabolically healthier older adults or in populations without obesity or metabolic syndrome. Although repeated ALM measurements strengthened the longitudinal assessment, the modest sample size may have limited statistical power, particularly for subgroup analyses, and constrains broader conclusions regarding biological aging trajectories. This study focused on DXA-derived ALM and did not include measures of muscle strength, gait speed, frailty, falls, or other functional outcomes; therefore, the findings should be interpreted as reflecting ALM preservation rather than sarcopenia or functional decline. Additional limitations relate to TL assessment. TL was measured using qPCR-based methodology, which provides a relative estimate of mean TL and is sensitive to pre-analytical and analytical sources of variability, including DNA extraction, storage, plate effects, calibrator selection, and laboratory conditions. Of note, intra- and inter-assay coefficients of variability were calculated and when the results were above 10%, the samples were reanalyzed. Moreover, leukocyte TL may be influenced by leukocyte-subset composition and should not be assumed to represent skeletal muscle TL, satellite-cell telomere dynamics, or muscle regenerative capacity [42–44]. Finally, because the cohort was embedded within the PREDIMED-Plus randomized clinical trial, the lifestyle intervention may have influenced body composition trajectories through an energy-reduced Mediterranean diet, physical activity promotion, and behavioral support for weight-loss [45]. Although treatment allocation was included as a covariate and no statistically significant interaction by study arm was observed, residual intervention-related effects cannot be excluded, particularly because TL was assessed only at baseline.

## Conclusion

In conclusion, longer baseline leukocyte TL was modestly associated with more favorable long-term ALM trajectories in older adults with overweight or obesity and metabolic syndrome. These findings should be interpreted cautiously given the observational design and the indirect nature of leukocyte TL as a systemic biomarker. Leukocyte TL may may reflect broader biological aging or cumulative biological stress related to muscle-mass trajectories, but it should not be interpreted as a direct marker of skeletal muscle telomere biology or regenerative capacity. Further longitudinal studies with repeated TL assessment, muscle function measures, and integrated biological profiling are needed to clarify the clinical relevance and mechanistic basis of this association.

## Supplementary information

Below is the link to the electronic supplementary material.

ESM 1 (DOCX 419 KB)

## Data Availability

Data described in the manuscript, codebook, and analytic code will be made available upon request pending application and approval of the PREDIMED-Plus Steering Committee. There are restrictions on the availability of data for the PREDIMED-Plus trial, due to the signed consent agreements around data sharing, which only allow access to external researchers for studies following the project purposes. Requestors wishing to access the PREDIMED-Plus trial data used in this study can make a request to the PREDIMED-Plus trial Steering Committee chair: jordi.salas@urv.cat. The request will then be passed to members of the PREDIMED-Plus Steering Committee for deliberation.
