# Supplementary material for: Telomere length and appendicular lean mass over six years in older adults with overweight or obesity and metabolic syndrome: a prospective cohort study
Source: GeroScience. 2026 Jun 20;48(6):7561–74. doi: 10.1007/s11357-026-02366-w (PMC13638864; doi:10.1007/s11357-026-02366-w)
Supplement: Supplementary file 1 [file 11357_2026_2366_MOESM1_ESM.docx]

| **Table S1.- Number of study participants with and without data on appendicular lean mass from centers having access to DXA device^a^** | | | |
| --- | --- | --- | --- |
| **Variable** | **Total** | **Included^b^** | **Non-included^c^** |
|  | **n** | **n (%)** | **n (%)** |
| **Recruiting center** |  |  |  |
| All centers with available DXA | 2,519 | 1,477 (58.6) | 1,042 (41.4) |
| Center 3 | 331 | 255 (77.0) | 76 (23.0) |
| Center 4 | 335 | 294 (87.8) | 41 (12.2) |
| Center 5 | 302 | 137 (45.4) | 165 (54.6) |
| Center 7 | 460 | 342 (74.3) | 118 (25.7) |
| Center 10^d^ | 628 | 93 (14.8) | 535 (85.2) |
| Center 20 | 205 | 130 (63.4) | 75 (36.6) |
| Center 23 | 258 | 226 (87.6) | 32 (12.4) |
| Abbreviations: DXA, dual energy x-ray absorptiometry.  ^a^Data are shown as numbers (percentages). The sample of 1,474 participants come from 7 out of the 23 PREDIMED-Plus recruiting centers, as those seven centers were the only centers that had DXA scanner available. In each of these seven centers, either all participants or a sub-sample were invited to DXA scans.  ^b^Participants with data on body composition measured with DXA and included in the analysis.  ^c^Participants without DXA data excluded from the analysis.  ^d^Center #10 was the vanguard center and started the recruitment and randomization many months earlier than the other centers, therefore many participants of center #10 had had their baseline evaluation before the DXA study started. | | | |

| **Table S2.- Number of study participants with and without data on appendicular lean mass from centers having access to telomere length measurements^a^** | | | |
| --- | --- | --- | --- |
| **Variable** | **Included** | **With ALM^b^** | **With ALM and telomere length^c^** |
|  | **n** | **n (%)** | **n (%)** |
| **Recruiting center** |  |  |  |
| All centers with available DXA | 1,088 | 435 (40.0) | 185 (42.5) |
| Center 7 | 460 | 342 (74.3) | 147 (43.0) |
| Center 10^d^ | 628 | 93 (14.8) | 38 (40.9) |
| Abbreviations: DXA, dual energy x-ray absorptiometry.  ^a^Data are shown as numbers (percentages). The sample of 1,088 participants come from 2 out of the 7 PREDIMED-Plus recruiting centers with DXA scanner available. In each of these two centers telomere length measurements were performed.  ^b^Participants with data on appendicular lean mass measured with DXA.  ^c^Participants with data on appendicular lean mass measured with DXA and available data on telomere length.  ^d^Center #10 was the vanguard center and started the recruitment and randomization many months earlier than the other centers, therefore many participants of center #10 had had their baseline evaluation before the DXA study started. | | | |

| **Table S3.- Baseline characteristics of study participants selected and non-selected for DXA measurements^a^** | | | | |
| --- | --- | --- | --- | --- |
| **Characteristic** | **Total population (n=6,874)** | **Selected^b^ (n=2,519)** | **Non selected^c^ (n=4,355)** | **P- value** |
| **Sociodemographic variables** |  |  |  |  |
| Age, mean (SD), years | 64.9 ± 4.9 | 65.4 ± 5.0 | 64.7 ± 4.9 | **0.001** |
| Female, No. (%) | 3335 (48.5) | 1178 (46.8) | 2157 (49.5) | **0.027** |
| Education level, No. (%) |  |  |  |  |
| Primary or less | 3362 (48.9) | 1212 (48.1) | 2150 (49.4) | 0.056 |
| Secondary | 1986 (28.9) | 770 (30.6) | 1216 (27.9) |  |
| College | 1526 (22.2) | 537 (21.3) | 989 (22.7) |  |
| Marital status, No. (%) |  |  |  |  |
| Single, divorced or separated | 897 (13.1) | 338 (13.4) | 559 (12.8) | 0.375 |
| Married | 5261 (76.5) | 1905 (75.6) | 3356 (77.1) |  |
| Widower | 716 (10.4) | 276 (11.0) | 440 (10.1) |  |
| Ethnicity |  |  |  |  |
| Caucasian | 6874 (100%) | 2519 (100%) | 4355 (100%) |  |
| African | 0 (0) | 0 (0) | 0 (0) | – |
| Asian | 0 (0) | 0 (0) | 0 (0) |  |
| Hispanic or Latino | 0 (0) | 0 (0) | 0 (0) |  |
| Other | 0 (0) | 0 (0) | 0 (0) |  |
| **Disease presence or medication usage at recruitment** | | | |  |
| Type 2 diabetes, No. (%) | 2121 (30.9) | 677 (26.9) | 1444 (33.2) | **0.001** |
| Hypercholesterolemia, No. (%) | 4813 (70.0) | 1652 (65.6) | 3161 (72.6) | **0.001** |
| Metabolic syndrome, No. (%) | 6874 (100.0) | 2519 (100.0) | 4355 (100.0) | – |
| Hypertension, No. (%) | 5758 (83.8) | 2126 (84.4) | 3632 (83.4) | 0.279 |
| Hypertriglyceridemia, No. (%) | 2862 (41.6) | 989 (39.3) | 1873 (43.0) | **0.002** |
| Low HDL, No. (%) | 2794 (40.7) | 1164 (46.2) | 1630 (37.4) | **0.001** |
| Hyperglycemia, No. (%) | 4572 (66.5) | 1665 (66.1) | 2907 (66.8) | 0.580 |
| Central obesity, No. (%) | 5575 (81.1) | 1989 (79.0) | 3586 (82.3) | **0.001** |
| Medication use, No. (%) |  |  |  |  |
| Insulin or other antidiabetic drugs | 1817 (26.4) | 536 (21.3) | 1281 (29.4) | **0.001** |
| Antihypertensive agents | 5378 (78.2) | 2013 (79.9) | 3365 (77.3) | **0.010** |
| Statins or other hypolipidemic drugs | 3560 (51.8) | 1249 (49.6) | 2311 (53.1) | **0.005** |
| **Lifestyle variables** |  |  |  |  |
| Physical activity, mean (SD), METs per minutes per day | 351.7 ± 328.5 | 382.4 ± 325.4 | 334.0 ± 329.1 | **0.001** |
| Sedentary time, mean (SD), hours per day | 6.0 ± 2.0 | 5.7 ± 1.9 | 6.2 ± 2.0 | **0.001** |
| Smoking status, No. (%) |  |  |  |  |
| Current smoker | 857 (12.5) | 318 (12.6) | 539 (12.4) | 0.341 |
| Former smoker | 2983 (43.4) | 1018 (44.4) | 1865 (42.8) |  |
| Never smoker | 3034 (44.1) | 1083 (43.0) | 1951 (44.8) |  |
| **Dietary variables** |  |  |  |  |
| Alcohol intake, mean (SD), g/d | 11.0 ± 15.0 | 11.9 ± 15.9 | 10.5 ± 14.5 | **0.001** |
| Total energy intake, mean (SD), kcal/d | 2361 ± 550 | 2373 ± 543 | 2354 ± 553 | 0.167 |
| Abbreviations: DXA, dual energy x-ray absorptiometry. erMedDiet, energy-restricted Mediterranean Diet. HDL, high-density lipoproteins. METs, metabolic equivalents SD, standard deviation.  ^a^Data are presented as n (%) or mean ± SD for categorical and continuous variables, respectively. Unpaired Student’s t-test and Chi-square tests were employed for continuous and categorical variables, respectively. Significant differences (P<0.05) were highlighted in bold type.  ^b^Participants selected for potential DXA measurements  ^c^Participants non-selected for DXA measurements | | | | |

| **Table S4- Baseline telomere length and appendicular lean mass over six years after excluding lost to follow-up participants (complete case analysis)^a^** | | | | |
| --- | --- | --- | --- | --- |
| **Variable** | **Multivariable-adjusted model** | | | |
|  | **Continuous** | **< median** | **≥ median** | **P-trend^b^** |
| **Appendicular lean mass (kg)** |  |  |  |  |
| Baseline | 0 (Ref.) | 0 (Ref.) | 0 (Ref.) | **0.014** |
| Year 1 vs baseline | 0.62 [–0.15 to 1.38] | 0 (Ref.) | 0.72 [–0.03 to 1.46] |  |
| Year 3 vs baseline | 0.70 [–0.06 to 1.47] | 0 (Ref.) | **0.74 [0.01 to 1.49]** |  |
| Year 6 vs baseline | **1.19 [0.42 to 1.95]** | 0 (Ref.) | **1.23 [0.48 to 1.98]** |  |
| **Upper limbs lean mass (kg)** |  |  |  |  |
| Baseline | 0 (Ref.) | 0 (Ref.) | 0 (Ref.) | **0.001** |
| Year 1 vs baseline | 0.31 [–0.06 to 0.68] | 0 (Ref.) | **0.39 [0.04 to 0.75]** |  |
| Year 3 vs baseline | **0.57 [0.20 to 0.94]** | 0 (Ref.) | **0.65 [0.29 to 1.01]** |  |
| Year 6 vs baseline | **0.51 [0.14 to 0.87]** | 0 (Ref.) | **0.56 [0.21 to 0.92]** |  |
| **Lower limbs lean mass (kg)** |  |  |  |  |
| Baseline | 0 (Ref.) | 0 (Ref.) | 0 (Ref.) | 0.135 |
| Year 1 vs baseline | 0.31 [–0.31 to 0.93] | 0 (Ref.) | 0.33 [–0.28 to 0.92] |  |
| Year 3 vs baseline | 0.13 [–0.48 to 0.75] | 0 (Ref.) | 0.10 [–0.50 to 0.70] |  |
| Year 6 vs baseline | **0.68 [0.06 to 1.30]** | 0 (Ref.) | **0.67 [0.06 to 1.27]** |  |
| Abbreviations: ALM, appendicular lean mass. CI, confidence interval.  ^a^Two-level linear mixed models were fitted with random intercepts at the cluster family (as couples from the same household were randomized together) and individual participants to assess the relationship baseline telome length, modeled as a continuous variable and by predefined categories (< median and ≥ median) and appendicular lean mass (outcome) measured repeatedly over time (at each follow-up visit and for the overall follow-up period). Multivariable-adjusted models included fixed effects for an interaction term between baseline telomere length and time, as well as age (years), sex (male or female), recruiting center, intervention group (control or intervention), education level (primary or less, secondary, or college), marital status (single, divorced or separated, married, or widowed), smoking status (former, or never), diabetes prevalence (yes or no), hypertension prevalence (yes or no), hypercholesterolemia prevalence (yes or no), physical activity (metabolic equivalents in minutes per day), sedentary time (hours per day), alcohol consumption in grams per day (and adding the quadratic term), and total energy intake (kilocalories per day). Lost to follow-up participants with no information for appendicular lean mass over time were excluded. Data are presented as mean differences [95% CI]. Significant values (*p*<0.05) were highlighted in bold type.  ^b^P-value for the overall follow-up period. | | | | |

| **Table S5.- Baseline telomere length and appendicular lean mass indexes after excluding lost to follow-up participants (complete case analysis)^a^** | | | | |
| --- | --- | --- | --- | --- |
| **Variable** | **Multivariable-adjusted model** | | | |
|  | **Continuous** | **< median** | **≥ median** | **P-trend^b^** |
| **Appendicular lean mass to height ratio** |  |  |  |  |
| Baseline | 0 (Ref.) | 0 (Ref.) | 0 (Ref.) | **0.022** |
| Year 1 vs baseline | 0.18 [–0.05 to 0.42] | 0 (Ref.) | 0.22 [–0.01 to 0.45] |  |
| Year 3 vs baseline | 0.22 [–0.02 to 0.45] | 0 (Ref.) | **0.23 [0.01 to 0.46]** |  |
| Year 6 vs baseline | **0.35 [0.12 to 0.58]** | 0 (Ref.) | **0.35 [0.13 to 0.58]** |  |
| **Appendicular lean mass to total body weight ratio** |  |  |  |  |
| Baseline | 0 (Ref.) | 0 (Ref.) | 0 (Ref.) | **0.011** |
| Year 1 vs baseline | 0.87 [–0.01 to 1.74] | 0 (Ref.) | **0.96 [0.11 to 1.81]** |  |
| Year 3 vs baseline | 0.67 [–0.20 to 1.55] | 0 (Ref.) | **0.92 [0.07 to 1.77]** |  |
| Year 6 vs baseline | **1.23 [0.35 to 2.10]** | 0 (Ref.) | **1.39 [0.54 to 2.24]** |  |
| **Appendicular lean mass to body mass index ratio** |  |  |  |  |
| Baseline | 0 (Ref.) | 0 (Ref.) | 0 (Ref.) | **0.009** |
| Year 1 vs baseline | 0.02 [–0.01 to 0.05] | 0 (Ref.) | **0.03 [0.01 to 0.05]** |  |
| Year 3 vs baseline | 0.02 [–0.01 to 0.05] | 0 (Ref.) | 0.02 [–0.01 to 0.05] |  |
| Year 6 vs baseline | **0.03 [0.01 to 0.06]** | 0 (Ref.) | **0.04 [0.02 to 0.07]** |  |
| Abbreviations: ALM, appendicular lean mass. CI, confidence interval.  ^a^Two-level linear mixed models were fitted with random intercepts at the cluster family (as couples from the same household were randomized together) and individual participants to assess the relationship baseline telome length, modeled as a continuous variable and by predefined categories (< median and ≥ median) and appendicular lean mass indexes (outcome) measured repeatedly over time (at each follow-up visit and for the overall follow-up period). Multivariable-adjusted models included fixed effects for an interaction term between baseline telomere length and time, as well as age (years), sex (male or female), recruiting center, intervention group (control or intervention), education level (primary or less, secondary, or college), marital status (single, divorced or separated, married, or widowed), smoking status (former, or never), diabetes prevalence (yes or no), hypertension prevalence (yes or no), hypercholesterolemia prevalence (yes or no), physical activity (metabolic equivalents in minutes per day), sedentary time (hours per day), alcohol consumption in grams per day (and adding the quadratic term), and total energy intake (kilocalories per day). Lost to follow-up participants with no information for appendicular lean mass indexes over time were excluded. Data are presented as mean differences [95% CI]. Significant values (*p*<0.05) were highlighted in bold type.  ^b^P-value for the overall follow-up period. | | | | |

**Fig. S1** **A priory** **interaction for the relationship between baseline telomere length as continuous and appendicular lean mass over six years with multiple baseline variables of the study.** Two-level linear mixed models were fitted with random intercepts at the cluster family (as couples from the same household were randomized together) and individual participants to assess the relationship between baseline telomere length as a continuous variable (exposure), and appendicular lean mass measured repeatedly over time (at each follow-up visit and for the overall follow-up period). Multivariable-adjusted models included fixed effects for an interaction term between baseline telomere length and time, as well as age (years), sex (male or female), recruiting center, intervention group (control or intervention), education level (primary or less, secondary, or college), marital status (single, divorced or separated, married, or widowed), smoking status (former or never), diabetes prevalence (yes or no), hypertension prevalence (yes or no), hypercholesterolemia prevalence (yes or no), physical activity (metabolic equivalents in minutes per day), sedentary time (hours per day), alcohol consumption in grams per day (and adding the quadratic term), and total energy intake (kilocalories per day). Data are presented as Beta standardized values [95% CI]. Significant values (*p*<0.05) were highlighted in bold type.
